# Addressing arbitrary choices of frequency band of interest in fNIRS hyperscanning

**DOI:** 10.1038/s41598-026-50540-z

**Published:** 2026-04-27

**Authors:** Xin Zhou, Florrie F. Y. Ng, Patrick C. M. Wong

**Affiliations:** 1 Brain and Mind Institute, The Chinese University of Hong Kong, Sha Tin, Hong Kong SAR China; 2 Department of Educational Psychology, The Chinese University of Hong Kong, Sha Tin, Hong Kong SAR, China; 3 Department of Linguistics and Modern Languages, The Chinese University of Hong Kong, Sha Tin, Hong Kong SAR, China

**Keywords:** fNIRS hyperscanning, Inter-brain connection, Frequency band of interest, Neuroscience, Psychology, Psychology

## Abstract

Neuroimaging hyperscanning—the monitoring of brain activity of two or more persons simultaneously—has emerged as a popular tool to uncover the neural mechanisms of social interactions. The use of functional near-infrared spectroscopy (fNIRS)—a non-invasive, child-friendly technique tolerant of motion artifacts—has significantly advanced the research of social interactions. Despite its popularity, the field has yet to agree on best practices for quantifying inter-brain connections (IBC) during social interactions, including the frequency band of interest (FOI) for signal analysis. Various choices of FOIs, along with subject-level physiological differences or experimental design, may have contributed to inconsistent findings across prior studies. In this study, we reviewed various methods used and their corresponding FOI results in previous fNIRS hyperscanning research focused on the topics of cooperation. Additionally, we propose a new methodology to quantify FOI that aims to point to the origin of synchronization between brains. We tested the proposed method on three independent fNIRS hyperscanning datasets. The three datasets involved three different populations and three types of social interactions commonly studied in the literature. We examined the effect of sample sizes and data exclusion rates on the calculation of FOIs and statistical results. We offer a method for testing and adoption within the fNIRS community, aimed at eliminating arbitrary FOI selections and potentially enhancing the reproducibility of results in future fNIRS hyperscanning research.

**Supplementary Information:**

The online version contains supplementary material available at 10.1038/s41598-026-50540-z.

## Introduction

Hyperscanning, by simultaneously monitoring brain activities between two or more people, has emerged as a hot approach to uncover the mechanisms underlying social interactions. Hyperscanning research analyzes inter-brain connections (IBC) to reveal how changes in one person’s brain activity align with the changes in another person’s brain activity and to quantify inter-personal brain synchronization. In this review, we concentrated on hyperscanning research by employing functional near-infrared spectroscopy (fNIRS), a non-invasive, child-friendly, and motion-tolerant neuroimaging technique^1^, which has become an essential tool for investigating the complexities of human social behavior in naturalistic settings^2^. One of the primary areas of focus within fNIRS hyperscanning research is the study of cooperative interactions^3^. This is because cooperation is a fundamental aspect of human social behavior and a social norm^4^. Prior fNIRS hyperscanning studies have revealed important information about the neural mechanisms that underpin cooperation and how social interactions can influence cognitive processes among different populations, including romantic partners^5^, parent–child dyads^6^, and schoolers with varying autistic traits^7^.

Despite its promise in studying social behaviors in real-world setting, previous studies have shown mixed findings regarding the synchronization of two brains as reviewed below. In addition, no consensus has been achieved to date on how to analyze fNIRS signal for hyperscanning research, with various methods being used to calculate IBC^8^. Specifically, prior studies have employed varied and arbitrary selections of the frequency band of interest (FOI) for calculating IBC, which complicates the comparison and may have contributed to the inconsistent findings. We will discuss these findings in detail below. Various methods were used to select the FOI in prior studies aiming to address the poor signal-to-noise ratios of fNIRS signals. The fNIRS Society has established best practices for fNIRS publications involving single-person research to improve signal-to-noise ratios^9^. However, single-person and hyperscanning fNIRS research differ in the metrics utilized to assess brain signals of interest based on the changes in hemoglobin data. The former computes task-specific cortical responses within individuals, whereas the latter focuses on the relation between two or more brains during naturalistic social interactions. Thus, the pipeline of signal preprocessing recommended for single-person fNIRS research may not be suitable for hyperscanning research.

The overarching goal of the current study is to address the arbitrary choices of FOIs and enhance the reproducibility of results in the fNIRS hyperscanning literature. To this end, we first reviewed the various rationales for selecting FOIs and the mixed FOI results in prior studies. Second, we propose an innovative methodology to select FOI based on the neurophysiology of the fNIRS signals when quantifying IBC. Specifically, we focus on the wavelet transform coherence (WTC) method to measure IBC^10^. IBC reveals how well changes in brain activity in one person align with changes in brain activity in another person across time and frequencies. This is the most widely used technique in fNIRS hyperscanning literature, with 70% of studies employing this method^8^. We provide guidance on the sample sizes required and channel exclusions to achieve stable statistical results.

### Review of cooperation studies using fNIRS hyperscanning

**Fig. 1:**
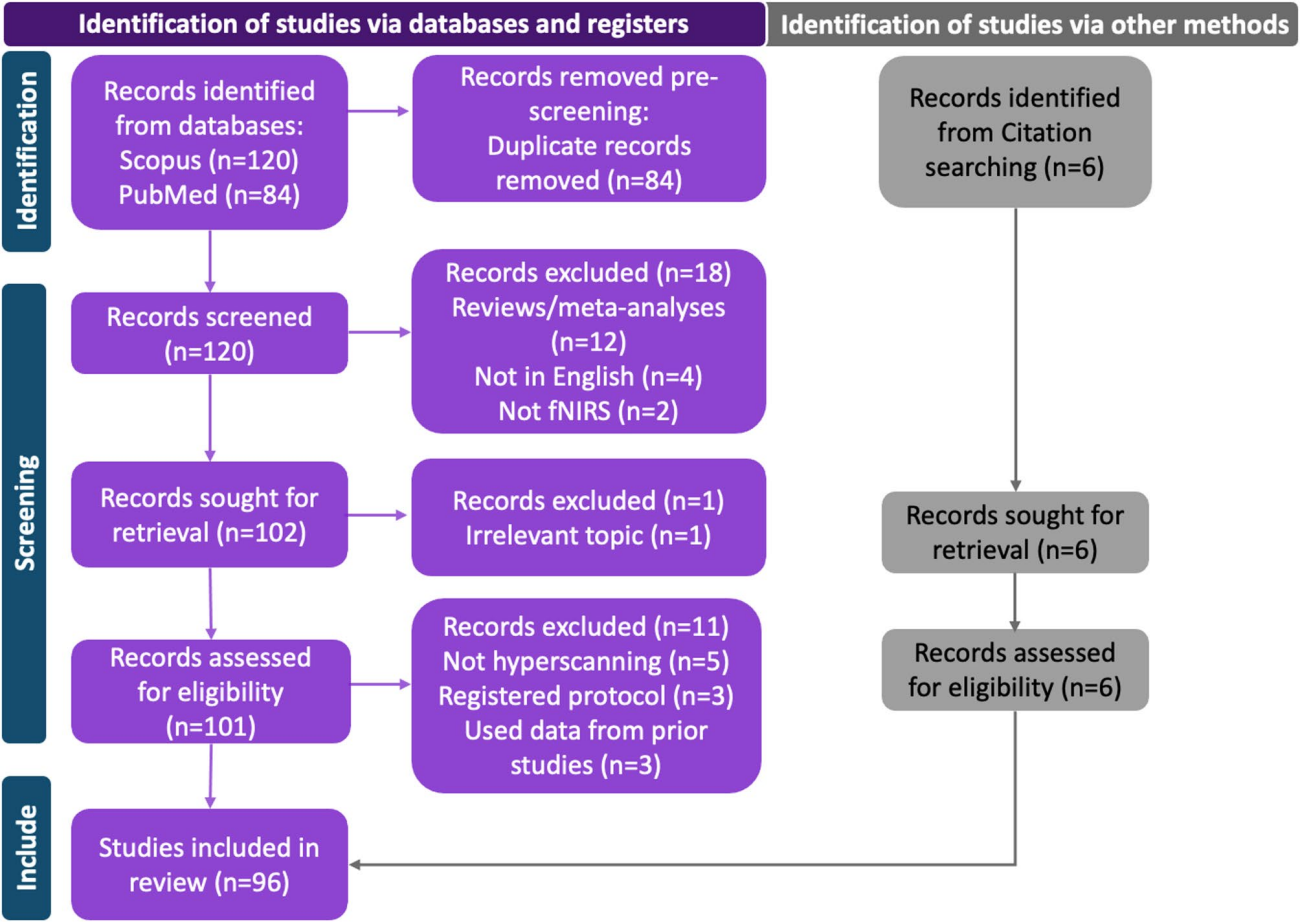
PRISMA flowchart of paper inclusions/exclusions.

We carried out a review according to the Preferred Reporting Items for Systematic Reviews and Meta-Analyses (PRISMA) guidelines^11^. Studies utilizing fNIRS hyperscanning techniques to investigate cooperation were discovered through searches on PubMed and Scopus, using the search terms: (“cooperation” OR “collaboration” OR “coordination”) AND (hyperscanning OR “two-person” OR “synchronization” OR “interbrain” OR “inter-brain”) AND (fNIRS OR NIRS OR “functional near-infrared spectroscopy”). We also identified a few references from citation search (Fig. 1).

### Various methods used to determine FOIs

**Fig. 2:**
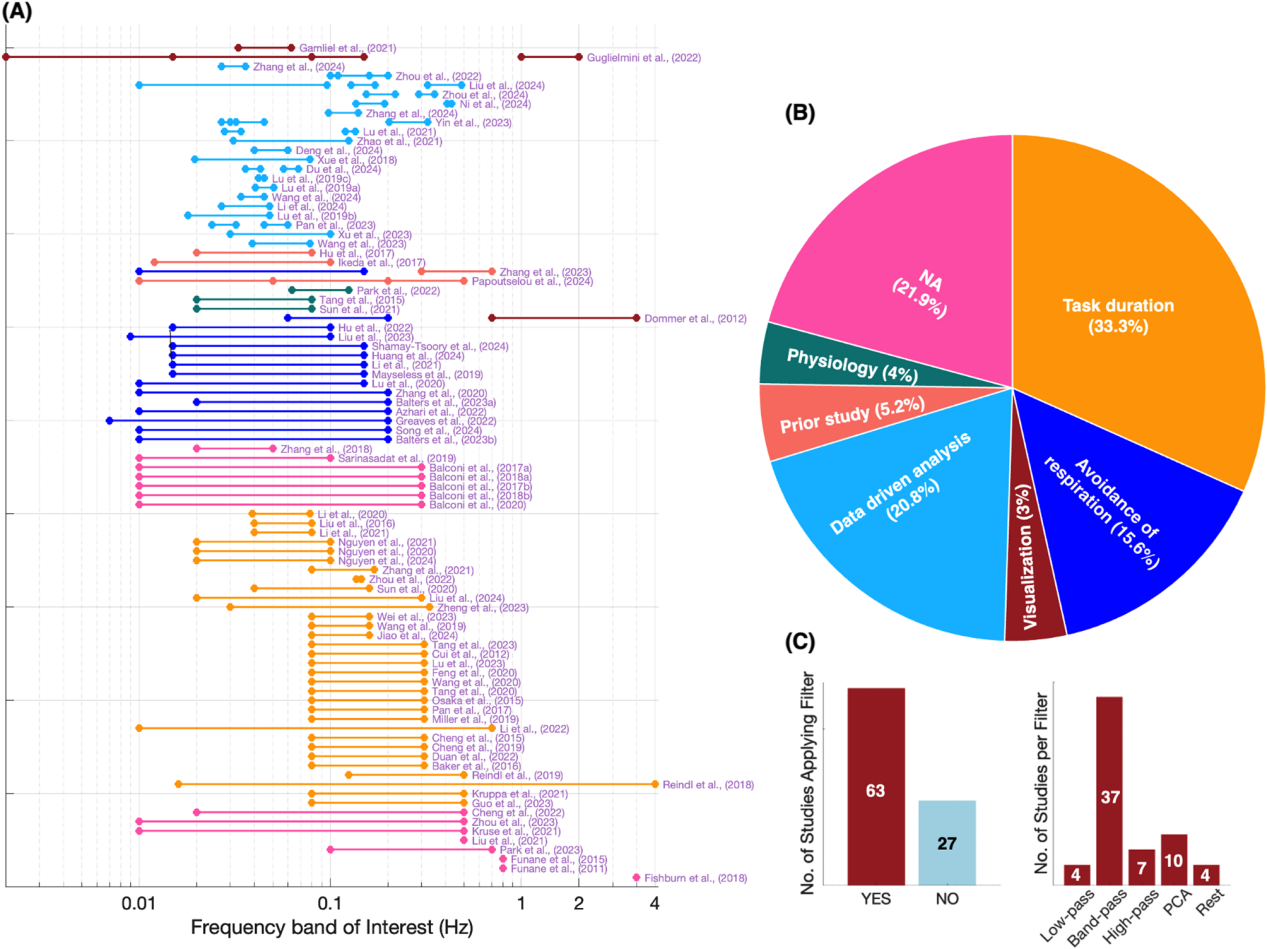
Varying frequency of interest (FOI) results in the prior fNIRS hyperscanning research about cooperation/collaboration.

As of November 2024, there have been 96 published fNIRS hyperscanning studies investigating the topics of cooperation, collaboration, and/or coordination (Fig. 1). Among the 96 studies, seven different approaches were used and researchers presumably adopted whatever approach they deemed most appropriate to decide the FOIs (Fig. 2). Specifically, 63 studies (65.6%) involved using a temporal or spectral filter to avoid confounds from the neurophysiological signals. Among those 63 studies, 15 only used a filter, while the rest combined filtering with one of below approaches to determine FOI. Among the 96 studies, 32 (33.3%) decided the FOI based on the duration of the trials or tasks; 20 (20.4%) utilized a data-driven analysis. Additionally, 12 studies chose the FOI based on visualization (n = 3), information from prior studies (n = 5), or the frequency of neurophysiological signals (n = 4). In 21 (21.9%) studies, the rationale for choosing FOI was not mentioned.

The first method utilized a temporal or spatial filter. Among the 96 studies, 63 (65.6%) reported applying a filter during signal pre-processing before calculating inter-brain connections. The reported filters included a low-pass (n = 4), band-pass (n = 37), high-pass (n = 7) filter, or a spatial filter based on principal component analysis (n = 10) to minimize the influence of neurophysiological signals like respiration, heartbeat, or Mayer waves (periodic changes in arterial blood pressure), and to reduce the effect of motion artefacts. The frequency ranges of the filters reported in previous studies included [~ 0.01, 0.2] Hz^12–16^, [0.02, 0.2] Hz^17^, [0.06, 0.2] Hz^18^, [0.015, 0.15] Hz^19–22^, [0.01 0.15] Hz^23,24^, and [~ 0.01 0.1] Hz^18,25^. An upper cutoff threshold set at 0.1 Hz was to further reduce the impact of Mayer waves (~ 0.1 Hz) on the calculation of IBCs^26^. The rationale for filtering high-frequency physiological signals has been well documented in single-person fNIRS studies^9^. However, they raise questions about the neural basis of IBCs. Specifically, would social interactions elicit synchronization in neurophysiological signals between interactive partners, regardless of whether they are strangers or different in ages? Moreover, as previously mentioned, naturalistic social interactions elicit neuronal responses to continuous and diverse external stimuli. Consequently, the responses of interest may significantly differ from those observed in single-person neuroscience studies, which typically reveal cortical responses to a few specific and periodic stimuli. Concentrating on investigations within a limited frequency range, such as below 0.2 Hz, may result in overlooking significant findings at higher frequencies that support social interactions, potentially leading to false negatives. Whereas 27 out of 96 studies specifically mentioned that no filtering was involved, because the WTC method by looking at the phase-locked signals between multiple brains is robust against motion artefacts and the neurological interference. In the rest 6 out of 96 papers, it was unclear whether a filter was used.

The method of determining the FOI based on task duration, which was the second most widely used approach and used by a third of the studies reviewed (Fig. 2), was first introduced in a study where pairs of participants engaged in computer-based cooperation versus competition tasks^27^. The trial durations ranged from 3.2 to 12.8 s, with resting breaks between trials, representing a traditional event-related design. The FOI was defined as the inverse of the task periods, calculated as 1/[12.8, 3.2], i.e., [0.08, 0.31] Hz, when assessing IBC using the WTC method^10^. Despite its popularity, this method can be challenging to implement, and the results can be difficult to interpret for four reasons. First, among studies using different tasks, different FOIs were also reported, for instance, [0.02, 0.3] Hz^28^ for an Etch-A-Sketch task, [0.03, 0.33] Hz^29^ for a pattern-board color-filling task, [0.08, 0.16] Hz^30^ for a cybermall task, [0.04, 0.16] Hz^31^ for an arithmetic operation task, ~ [0.04, 0.08] Hz^32–34^ for build-up (e.g., Jenga tower) and joint-drawing games, [0.02, 0.1] Hz^35–37^ for parent–child problem-solving tasks, and [0.137, 0.145] Hz^38^ for a Jigsaw puzzle game. Even when using the same computer-based task^27^ or a modified version, various FOIs were reported, including [0.016, 5] Hz^6^, [0.08, 0.5] Hz^39,40^, [0.125, 0.5] Hz^41^, [0.08, 0.31] Hz^5,27,42–53^, and [0.08, 0.16] Hz^54–56^, in correspondence with varying task durations. Second, the concept of a trial is often inapplicable to continuous social interactions in ecologically valid settings where the task formats are substantially different from those in traditional event-related designs. For instance, a lecture in a real-world classroom is a composite of interrelated events, the duration of which is not easily defined. Third, the hemodynamic response function (HRF) is not perfectly elicited by external stimulation, and so whilst FOI may approximately be the inverse of the task duration, it may not be exact, challenging the underlying premise of this method. Fourth, naturalistic social interactions encompass a variety of external stimuli, resulting in changes in hemodynamic responses that would be a convolution of multiple HRFs with various periods, rather than a fixed period of identical stimulation.

The third most common approach is the data-driven analysis method, which has become quite popular in the last few years and involves three specific steps. A WTC would first be used to calculate the IBC between two participants across frequencies and time points. Then, a bin-by-bin analysis would be conducted across multiple experimental conditions, against the baseline control condition for each frequency bin or a randomly generated null distribution. The FOI was identified as any three or more consecutive frequency bins that demonstrated significance, or any frequency bins that showed significance after applying a multiple comparison statistical control method. Finally, hypotheses would be tested on the identified FOIs. Although this method seems beneficial for uncovering study-specific IBCs, four major issues have been observed. First, to simplify the multiple comparison correction process when conducting bin-by-bin analyses, a few studies investigated IBCs within specific initial frequency ranges by applying filters in signal pre-processing, instead of testing the hypothesis across the entire frequency range. Among the 20 studies that determined FOIs using the data-driven analysis (Fig. 2), three reported initial frequency ranges of [~ 0.01, 1] Hz^57–59^, six studies in [~ 0.01, 0.7] Hz^60–65^, two studies in [0.01, 0.5] Hz^7,66^, one in the range of [0.01, 0.3] Hz^67^ and [0.01, 0.2] Hz^68^, and four studies below 0.1 Hz^69–72^. Because bin-by-bin data-driven analyses depend on fNIRS signals, varying initial frequency range selections might have led to inconsistent results in previous studies, particularly when sample sizes were small or signal-to-noise ratios were suboptimal. Second, this method raises the potential concern of double-dipping or circularity^73^, as it uses the same dataset for both FOI selection and selective analysis. Consequently, the resulting statistics are not inherently independent of the selection criteria. For instance, in a study that examined the differences in IBC in triads of students who learned poems cooperatively or independently^69^, the FOI was chosen by computing the differences in IBC between the two learning conditions and further compared with a null distribution. The increases in IBC from independent to cooperative learning were then correlated with their communicative behaviors during the cooperation. As the differences in IBC between the two conditions—cooperative and independent learning of idioms were strongly tied to the communicative behaviors in the cooperative learning mode, such correlational analyses were susceptible to circularity. Third, past studies that used this methodology have reported very different FOIs and often with smaller ranges of FOI (mean: 0.042 Hz, 95% CI [0.035, 0.049] Hz), compared to the FOI from studies determined based on task duration (Fig. 2). Among the 20 studies, 8 identified two or three FOIs of significance per task^7,57,59,63,64,66,69,74^, which introduced complexities in further hypothesis testing and made result dissemination challenging. Fourth, depending on the contrasts—whether between experimental conditions with different manipulations or compared with a rest session involving minimal social interaction between participants—the bin-by-bin data-driven analysis may yield different results within the same study and across different studies as discussed above.

### An innovative approach to determine the FOIs

To tackle the issues mentioned above regarding the arbitrary selection of FOI for fNIRS hyperscanning research, the current study proposes an innovative method based on the neurophysiology of fNIRS signals rather than driven by task durations or specific hypotheses. fNIRS uses a back-reflection measurement from a light source to an adjacent detector at about 30 mm, providing a regular fNIRS channel. As near-infrared light travels in and out of the extracerebral tissue, it passes through highly vascularized regions, including the scalp and skull, which have an average total thickness of about 13 mm^75^. fNIRS is very sensitive to the responses in extracerebral tissues and comparatively less sensitive to changes in hemoglobin in the deeper cerebral tissue^75^. The fNIRS signals comprise stimulus-evoked and non-stimulus-evoked, systemic and neuronal responses from the extracerebral and cerebral tissues^76^. The systemic responses comprise concentration changes in hemoglobin related to cardiac activity, respiration, changes in blood pressure, and vasomotion^77^. Signals of interest to researchers—concentration changes in hemoglobin associated with neuronal activity due to neurovascular coupling—are exclusive to the cerebral tissue and represent a minor part of the overall fNIRS data. In single-person fNIRS studies, current best practices recommend employing short channels (about 8 mm long) that are short enough to access extracerebral but not cerebral tissues^9,78^. Signals recorded from short channels have been demonstrated to successfully reduce the extracerebral components from regular channels, to improve the ratio of task-related neuronal signals of interest to noise^76,79^. When assessing IBCs during social interactions in multi-person neuroscience, we also aim to observe the synchronization between two or more brains associated with neuronal activity.

In this study, we propose to compare the frequency of signals measured between the regular and short channels. The frequency components that showed significantly greater IBC in the regular channels compared to the short channels should indicate the concentration changes in hemodynamic responses associated with neuronal activity, which are not detectable by the short channels. This method offers an innovative approach to investigating the origins of neural synchronization in multi-person contexts, mitigating the risk of false findings that plagued earlier studies due to their methodological restrictions. We tested this methodology on three independent datasets from three fNIRS hyperscanning experiments, each involving different types of social interactions across various populations. We hope this method would identify FOI independently of the hypothesis to test (for specific types of social interaction), thus avoiding the double-dipping issue faced by some previous methods.

## Methods

### Participants

Experiment 1 involved fifty-eight triads of participants, with one instructor and two school-aged children per triad. Each instructor participated in at least one session, involving 24 instructors (mean ± SD of age: 34.9 ± 9.8 years, 4 males) and 116 students (mean ± SD of age: 8.2 ± 0.5 years, 64 males). Experiment 2 involved fifty-five dyads of mothers (mean ± SD of age: 42.1 ± 4.3 years) and their children 9–12 years of age (mean ± SD of age: 9.8 ± 0.9 years; 31 females). Experiment 3 involved forty-four dyads of young adults (38 males and 50 females, mean and standard deviation (SD) of age: 20.6 ± 1.5 years). All three studies were approved by the Joint Chinese University of Hong Kong–New Territories East Cluster Clinical Research Ethics Committee. The study protocols were carried out following the Declaration of Helsinki. All participants (or parents of the child participants) provided written consent and were reimbursed for their participation.

### fNIRS data acquisition

In Experiment 1, an adult instructor taught two school-aged children mathematics (i.e., friction and perimeter) in two separate sessions. Each session started with a rest condition, followed by a pre-test, a lecture, and a post-test condition. Data from the rest and lecture conditions were reported in the current study. In Experiment 2, children performed a map task and story-telling task independently (with no interaction with their mothers) and then together with their mothers. In Experiment 3, dyads of college students engaged in a dual-task paradigm across four sessions. The primary task involved playing a Jenga game, while the secondary task required them to listen to Cantonese stories with varying levels of background noises. For an illustration of the three study paradigms and a more detailed explanation of the three experiments, please see the supplementary materials and Fig. S1.

**Fig. 3:**
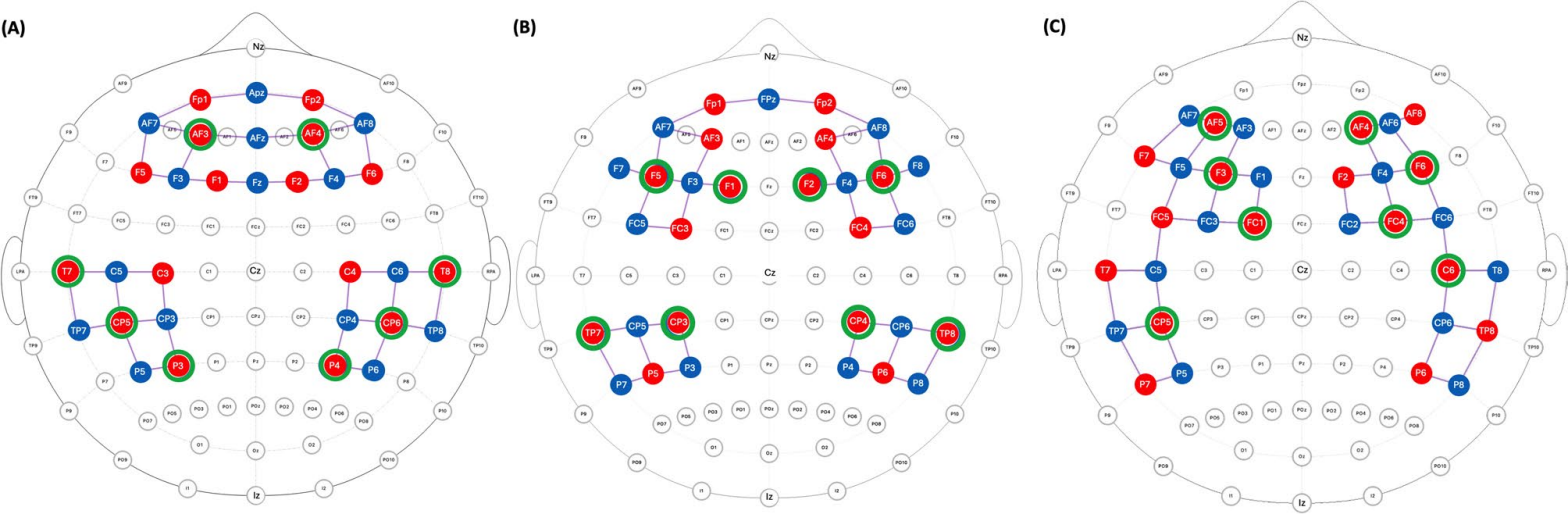
fNIRS montages three experiments. Panels (**A**), (**B**), and (**C**) plot the montage in experiments 1, 2, and 3, respectively. Red, blue, and green circles represent light sources (n = 16), detectors (n = 15), and short-channel detectors (n = 8), respectively. The red circles within the green circles represent short-channel sources.

The fNIRS data were collected using one continuous-wave NIRS instrument (NIRSport2 devices, NIRx medical technologies, LLC) per participant. Each device had 16 LED light sources and 16 avalanche photodiode (APD) detectors, with a sampling frequency of 5.08 Hz. Each LED light source emitted near-infrared light with wavelengths of 760 nm and 850 nm. A light source paired with detectors located at about 30-mm distance provided regular fNIRS channels that collected signals. Experiment 1, Experiment 2, and Experiment 3 included 42, 36, and 40 regular channels per participant, respectively. In addition, 8 short-channel detectors were individually connected to 8 light sources, resulting in 8 short channels per person at an approximate distance of 8 mm (Fig. 3). The three studies used different montages to address study-specific interests (Fig. 3); however, the three montages overlapped much as they all covered the social attention network—the bilateral fronto-parietal network— where inter-brain synchrony has often been reported in the hyperscanning literature.

### fNIRS data analysis

**Fig. 4:**
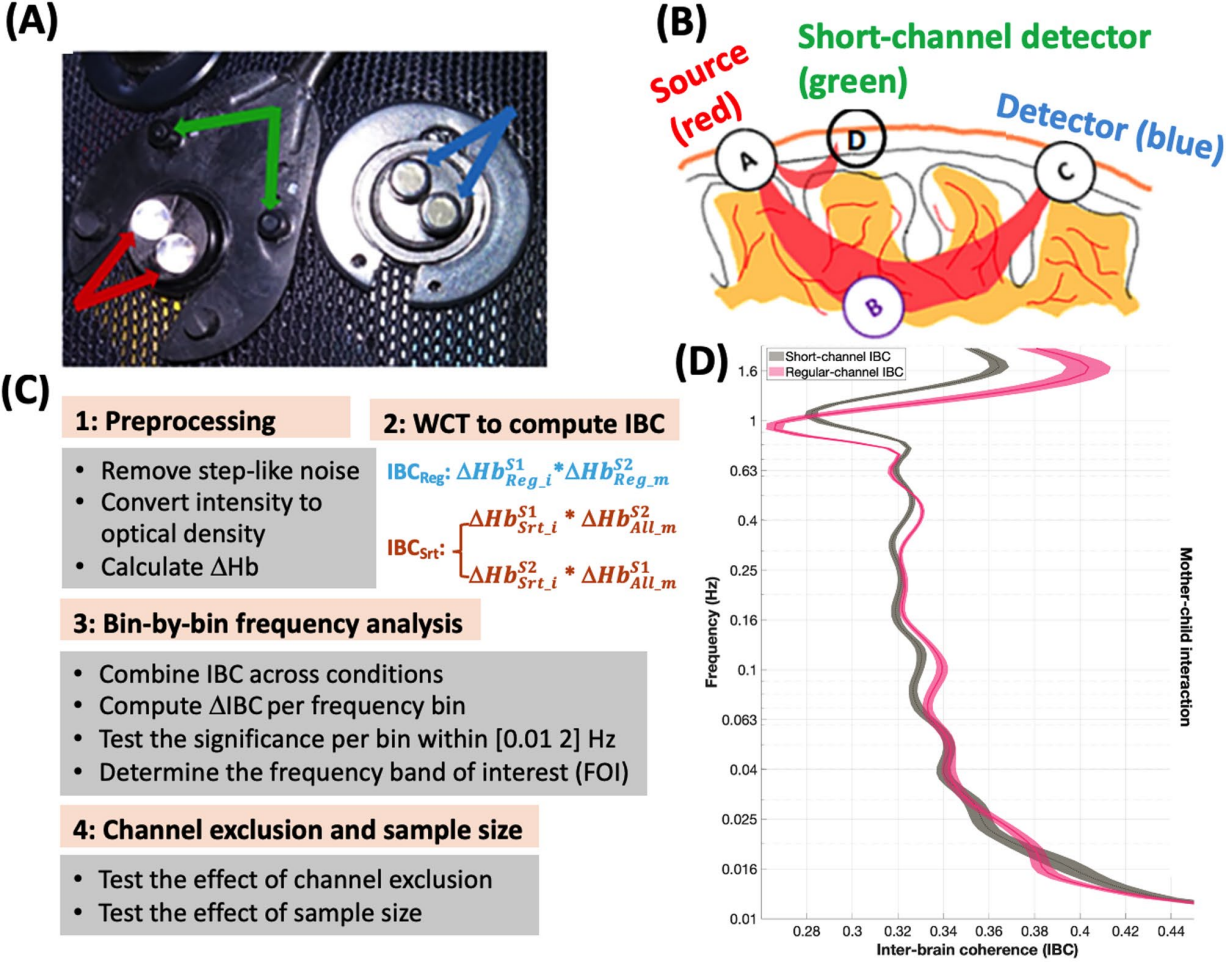
Signal processing pipeline. Panels (**A**) and (**B**) show an example of the short-channel detector and its connection, adapted from our prior study^79^. Panel (**C**) shows the example pipeline of signal preprocessing, the calculation of inter-brain connections (IBC), and the examination of frequency of interest (FOI) for fNIRS data. The IBC between two regular channels (IBC_Reg_) was computed as the wavelet transform coherence (WTC) between fNIRS measures (ΔHbO and ΔHbR) from a regular channel attached to a short-channel source (Reg_i; Fig. 3) in subject 1, i.e., ${\Delta Hb}_{Reg\_i}^{S1}$ and fNIRS measures from another regular channel attached to a short-channel source (Reg_m) in subject 2, i.e., ${\Delta Hb}_{Reg\_m}^{S2}$. The IBC values for short channels (IBC_Srt_) were computed as the WTC between any short channel in subject 1 (${\Delta Hb}_{Srt\_i}^{S1}$) with all regular channels attached to short-channel sources in subject 2 (${\Delta Hb}_{All\_i}^{S1}$), and between any short channel in subject 2 (${\Delta Hb}_{Srt\_i}^{S2}$) with all regular channels attached to short-channel sources in subject 1 (${\Delta Hb}_{All\_i}^{S1}$). Panel (D) plots the group mean (solid lines) and standard error of means (shaded areas) of the IBC in the regular (pink) and short (grey) channels.

The fNIRS data were imported into and analyzed in MATLAB (The MathWorks, Natick, MA) with scripts from HOMER2 software^80^ and written by the authors to preprocess data, involving the following four steps (Fig. 4C). The selection of FOI utilized short channels of 8 mm (Fig. 4A, B), which were specifically designed to penetrate only to the depth to measure cortical responses from extracerebral tissues^78^.

In Step 1, signal pre-processing was conducted consisting of 1) removing step-like noise, 2) converting light intensity to optical density, and 3) calculating ΔHbO and ΔHbR with age-dependent differential pathlength factors (DPFs) being used, using MATLAB code from Homer2 toolbox. No filtering or other denoising method was involved in the pre-processing step, as the WTC method was quite robust to motion artifacts^27^. Further, an inappropriate choice of filters may introduce distortion to the data. We refrained from pre-selecting any specific frequency range to ensure we did not overlook significant results. Further, our analyses focused on the IBC between the oxygenated hemoglobin (ΔHbO) data but not the deoxygenated hemoglobin (ΔHbR). This was because our prior study showed that IBC values calculated from the ΔHbO and ΔHbR data were highly correlated^7,81^, and most previous fNIRS hyperscanning research has reported IBC results calculated from the ΔHbO data. Reporting the same measures enhances the comparability of our findings with those in past studies.

In Step 2, we examined the differences in IBC between regular and short channels, focusing on the eight light sources that were connected with short-channel detectors, i.e., short-channel sources, each connected to 2–4 regular detectors (blue circles; Fig. 3). We included all short channels that were widespread on the montage to capture area-general and area-specific responses from the extracerebral tissue; we focused on the short-channel sources, instead of all light sources, to have comparable representations between the regular and short channels. To calculate the IBC between participants’ regular channels, we first computed the WTC between the regular channels linked to a short-channel source on one participant and those linked to a short-channel source on the other. The WTC values—a time–frequency matrix for each channel pair were averaged across time per frequency bin. We then computed the WTC of the two short channels (one from each participant), averaged across time per frequency bin, and subtracted them from all pairs of regular channels connected with the same short-channel sources in the two participants. Step 2 results in ΔIBC across frequency bins.

In Step 3, a one-sample t-test was conducted on ΔIBC across channels and dyads of participants for each frequency bin within the frequency range of 0.01–2 Hz, with a total of 92 frequency bins. For each dyad, experimental data from multiple conditions of the same task were combined, assuming that the neuronal signals driving the synchronization between two or more brains would share the same FOI, and may or may not differ in amplitude across these conditions. A Bonferroni method was used for multiple comparison corrections (n = 92 bins)^82^. The FOI was then defined as the range where three or more consecutive frequency bins exhibited greater than zero ΔIBC with adjusted *p*-values < 0.05 at the group level. Selecting three or more consecutive frequency bins was aimed at minimizing false positives.

In Step 4, we further investigated the impact of fNIRS data quality and sample size on the estimation of FOI and the subsequent statistical outcomes of IBC analysis. To quantify fNIRS signal quality, the scalp coupling index (SCI)—the correlation between heartbeat signals recorded from the two wavelengths for each fNIRS channel was computed per channel. An SCI of 1 signifies a perfect correlation between the fNIRS signals measured at the two wavelengths, indicating excellent signal quality. However, it is unknown whether and how signal quality and channel exclusion affect the IBC results in hyperscanning research. To address this issue, we systematically investigated the FOI by excluding channels at various SCI cutoff thresholds. The SCI cutoff values were calculated at each percentile of SCI values across all participants and sessions. With a SCI cutoff threshold of 100^th^ percentile, all the channels were excluded from this step of analysis. We assessed the impact of sample size by calculating the FOI across a range of sample sizes (from 2 to 40 dyads, in steps of 2), using the data from Experiment 2 and Experiment 3, both of which consisted of dyadic data with smaller sample sizes than Experiment 1. For each sample size, we randomly selected the number of dyads from the pool and repeated the above steps for FOI calculation 500 times. We assessed robustness for each sample size and FOI by requiring that the frequency bins show significance in more than 475 of the 500 repetitions (exceeding a 95% threshold) at the 95% confidence level.

## Results

### The frequency band of interest (FOI) results

Across the three experiments, the regular channels showed greater IBC results than the shorter channels in 3 or 4 comparable FOIs. For each FOI per task for each experiment, the significance of the FOI, e.g., Cohen’s d, 95% confidence interval (CI), and adjusted p values after multiple comparisons were summarized in Table 1. Across all studies with various tasks, the regular channels showed greater IBC in the high-frequency FOI of [1.03, 2] Hz, which corresponds to the frequency range of heart rate signals. Experiment 1 and Experiment 3 (in the interactive sessions) showed a wide mid-frequency FOI of starting from 0.073 to around 0.5 Hz. Experiment 2 (in the independent sessions) and Experiment 3 showed increased IBC in two FOIs, which covered a mid-frequency range similar to the one above but were separated by a gap between 0.163 and approximately 0.22 Hz. All sessions except the interactive session in Experiment 2 showed increased IBC in the very-low frequency FOI, i.e., under 0.04 Hz.

**Table 1:** Summary of the study information and statistical results for the frequency band of interest (FOI) in each study.

Experiment 1: 56 triads (1 teacher- 2 students)				
Task	Rest		Lecture	
Conditions & durations	N = 2 (2 min each)		N = 2 (5–10 min each)	
FOI & significance	[1.03 2] Hz	*d* = 0.72, 95% CI = 0.71; *p* < 10^–6^	[1.03 2] Hz	*d* = 1.19, 95% CI = 1.18; *p* < 10^–6^
	[0.073 0.435] Hz	*d* = 0.54, 95% CI = 0.53; *p* < 10^–6^	[0.073 0.73] Hz	*d* = 0.58, 95% CI = 0.57; *p* < 10^–6^
	[0.024 0.031] Hz	*d* = 0.14, 95% CI = 0.13; *p* < 10^–6^	[0.014 0.019] Hz	*d* = 0.11, 95% CI = 0.10; *p* < 10^–6^
Experiment 2: 55 dyads (mother—child)				
Task	Independent		Interactive	
Conditions & durations	N = 2 (5 min each)		N = 2 (5 and 8 min)	
FOI & significance	[1.03 2] Hz	*d* = 0.68, 95% CI = 0.66; *p* < 10^–6^	[1.03 2] Hz	*d* = 0.88, 95% CI = 0.86; *p* < 10^–6^
	[0.218 0.435] Hz	*d* = 0.39, 95% CI = 0.37; *p* < 10^–6^	[0.073 0.489] Hz	*d* = 0.40, 95% CI = 0.38; *p* < 10^–6^
	[0.082 0.163] Hz	*d* = 0.35, 95% CI = 0.33; *p* < 10^–6^		
	[0.020 0.024] Hz	*d* = 0.09, 95% CI = 0.07; *p* < 10^–6^		
Experiment 3: 44 dyads (university students)				
Task	Dual-task: Jenga play and story listening			
Conditions & durations	N = 4 (~ 6 min each)			
FOI & significance	[1.03 2] Hz	*d* = 1.17, 95% CI = 1.15; *p* < 10^–6^		
	[0.259 0.435] Hz	*d* = 0.35, 95% CI = 0.33; *p* < 10^–6^		
	[0.073 0.163] Hz	*d* = 0.46, 95% CI = 0.44; *p* < 10^–6^		
	[0.019 0.039] Hz	*d* = 0.17, 95% CI = 0.15; *p* < 10^–6^		

### The effect of channel exclusion and sample size

**Fig. 5:**
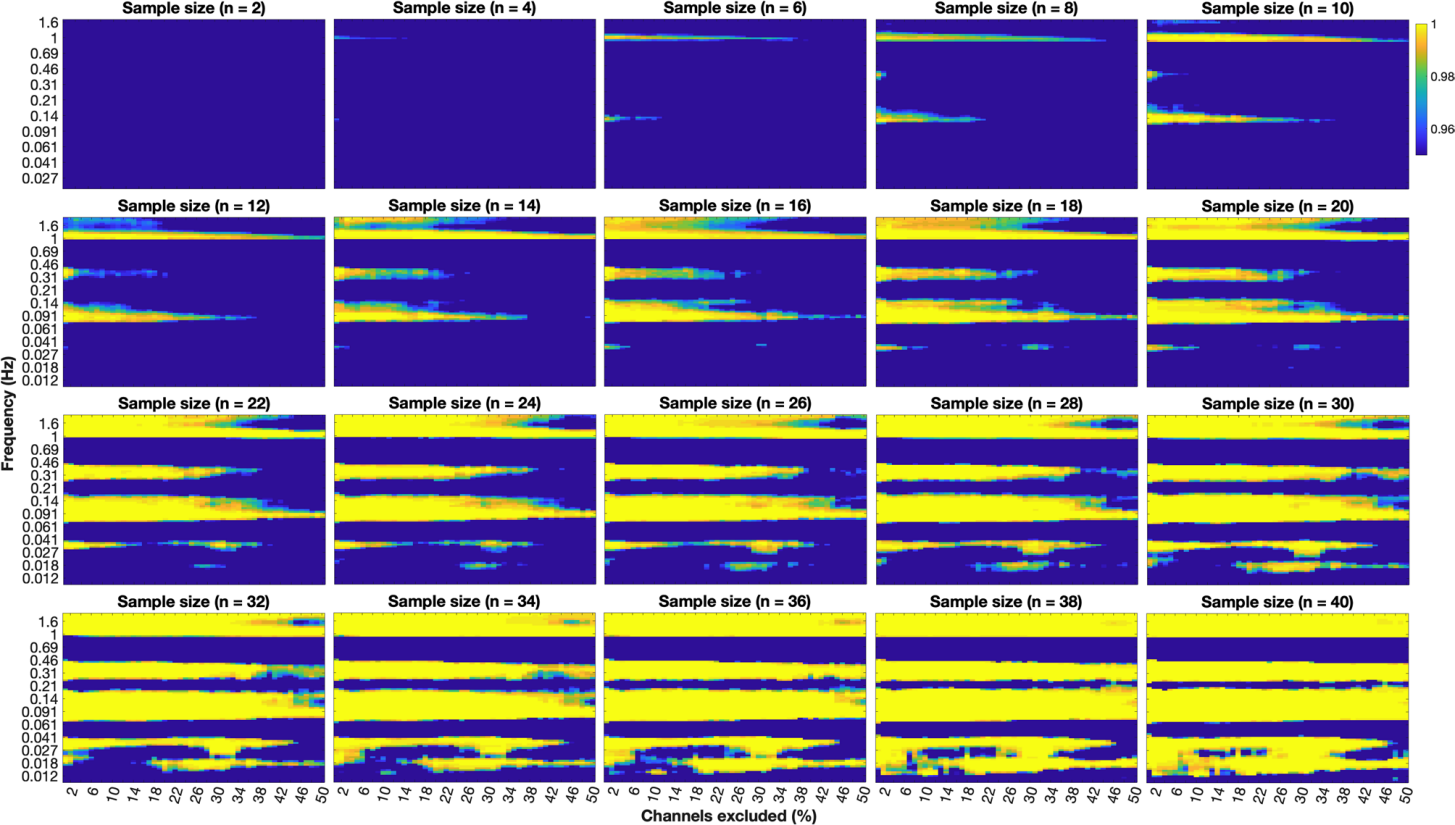
Significance of FOI across various channel exclusion rates and sample sizes. This plot was based on data from experiment 3. Yellow colors indicate robustness above 95% among 500 repetitions.

To examine the impact of sample size and channel exclusion, we computed the robustness of the significance based on 500 repeated iterations for every sample size and each exclusion rate for both Experiment 2 and Experiment 3. The robustness results from Experiment 3 are shown in Fig. 5; yellow colors represent that regular channels showed greater IBC than the short channels, with robustness exceeding a 95% threshold. As the sample size increased, three to four FOIs emerged that showed consistent results (see Table 1). As the exclusion rate increased, the robustness of the FOI decreased, this was particularly the case when sample sizes were small. Robustness results stabilized once the sample size reached a sufficient threshold (e.g., n ≥ 32). Similar results were observed in Experiment 1 (see supplementary materials Fig. S2).

**Fig. 6:**
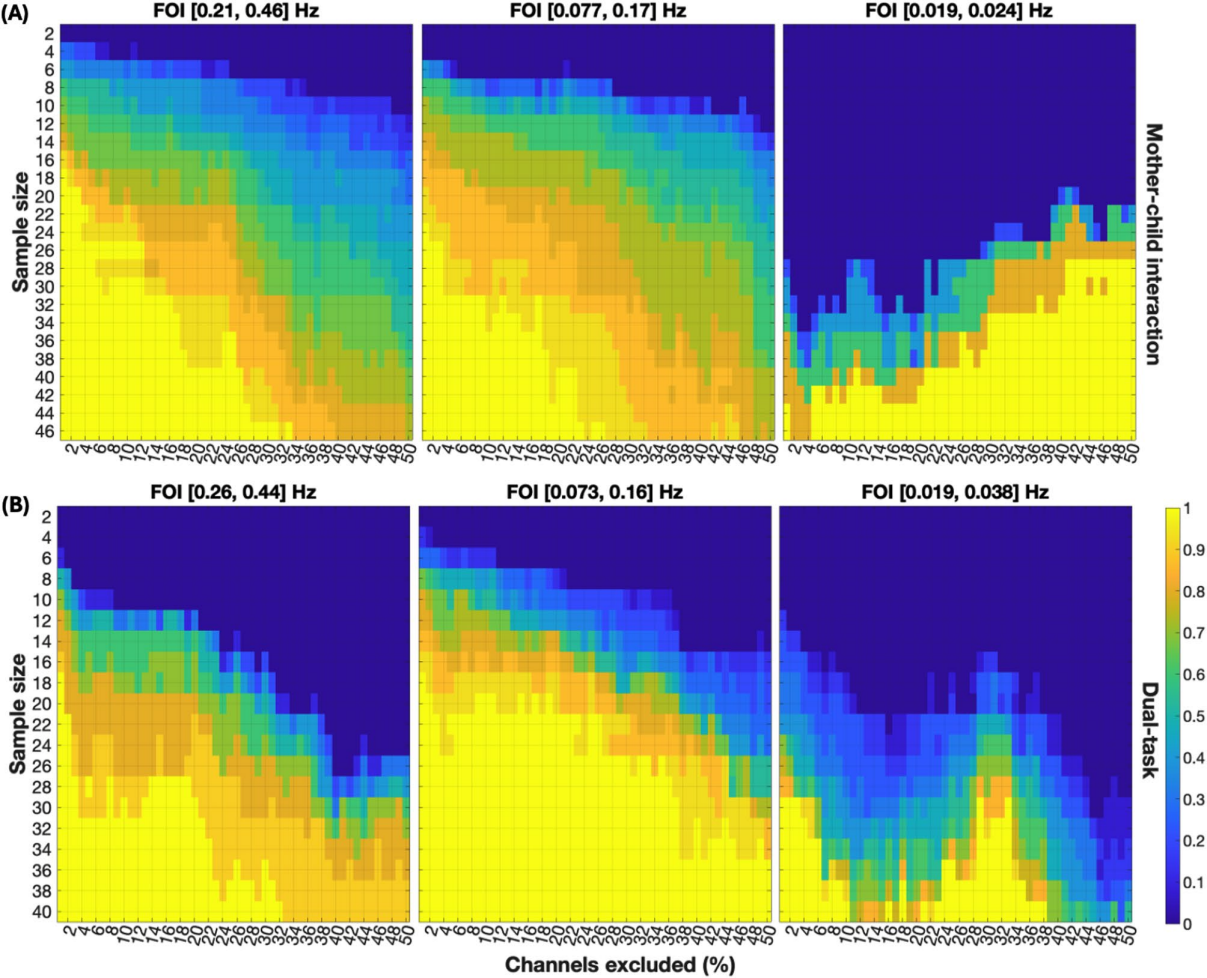
Significance at the FOI level. Panels (**A**) and (**B**) plot the results from Experiment 2 and Experiment 3, respectively.

To pinpoint the sample size required and an optimal channel exclusion rate, we focused on the FOIs below 0.5 Hz for Experiment 2 and Experiment 3. For each FOI, we measured how many robust frequency bins (exceeding the 95% threshold) remained in each cluster as we changed the sample size and exclusion rate. As shown in Fig. 6, for both Experiment 2 (Fig. 6A) and Experiment 3 (Fig. 6B), for the two FOIs between ~ [0.21 0.46] Hz and ~ [0.07 0.17] Hz, with the sample size increasing to 32, the number of frequency bins within each FOI that demonstrated significance stabilized. With such a sample size, channel exclusion rate increasing did not alter the number of frequency bins within the two FOIs until 10%. With the channel exclusion rate increasing further, the sample size required to maintain a high robustness undoubtedly increased. In contrast, for the low-frequency FOI of [0.019 0.038] Hz, various channel exclusion rates resulted in quite different numbers of frequency bins remaining significant. The statistical results of this FOI remained relatively stable for an exclusion rate between 5 and 7% for both studies. Similar results were found in Experiment 1. Please see the supplementary materials for the results (Fig. S3).

## Discussion

Previous fNIRS hyperscanning research has reported inconsistent findings of IBC during social interactions, likely due to the varying selections of FOIs and various ways to compute IBC. To date, no consensus has been reached regarding the rationale or method for determining the FOI in fNIRS hyperscanning research. The current study proposes a new methodology for selecting FOIs to calculate IBC in fNIRS hyperscanning research. This method utilizes short channels, which are recommended as part of current best practices in single-person fNIRS research to reduce noise signals, such as systemic responses from extracerebral tissue, and enhance neuronal signal-to-noise ratios^9,79^. This method where neural synchronization during social interactions is supposed to originate, potentially paving the way for utilizing fNIRS hyperscanning techniques in multi-person neuroscience.

### The neurophysiology driving the inter-brain coherence within four FOIs

We evaluated the proposed method using three independent datasets that featured distinct samples from different age groups and three types of social interactions: instructor-learner interactions involving young children in Experiment 1, mother–child interactions in Experiment 2, and cooperation among young adults in Experiment 3. The results revealed four comparable FOIs across the three experiments.

The greater IBC between the regular channels compared to the shorter channels in the relatively high FOI—[1 2] Hz—likely indicates similarities in heart rate signals between participants. As heart rate signals are the dominant signals in fNIRS data, and our data comprised children above 9 years of age and adults who have comparable heart rates, it is not surprising that we observed IBC between them when co-present in the same room and/or performing the same tasks. To dive into the cardiac dynamics during social interaction, we extracted heartbeat signals, calculated inter-beat-interval signals, and computed inter-beat-interval coherence between dyads of college students in Experiment 3. Our results found that regular channels also showed greater inter-beat-interval coherence in frequency ranges that correspond to heartbeat and Mayer waves, suggesting an increase in synchrony in both neuronal responses (i.e., inter-brain coherence) and associated cardiac activity (inter-beat-interval coherence) within the cerebral issue between two interactive participants during social interactions. Future studies may also need to include relatively higher frequencies in the analysis to examine the cardiac dynamics for hyperscanning research. Please detailed description and results in the supplementary materials (Fig. S4).

For the mid-frequency FOI—[0.22, 0.49 Hz]—across the three experiments, and up to 0.7 Hz in Experiment 1 during the lecture conditions, greater IBC could be due to three reasons. The greater IBC in this FOI could be first and most likely attributed to verbal communication between interaction partners, which was present across three experiments in the interactive tasks. In line with this result, two prior studies that involved instructor-learner verbal communications also reported increased IBC in relatively high-frequency bins^83,84^. Both studies employed bin-by-bin data-driven analyses to identify FOIs by comparing the teaching sessions to a resting state. The first study involved an instructor teaching psychological concepts using two strategies: explanation and scaffolding^83^ and reported increased IBC in an FOI of [0.45, 0.57] Hz^83^. The second study involved an instructor teaching numerical concepts using three teaching styles: lecturing, interactive methods, and pre-recorded videos^84^; it identified two significant FOIs—[0.5, 0.7] Hz that was related to teaching outcomes, and [0.3, 0.4] Hz that was associated with teaching styles^84^. These results were comparable with the results reported in the current study. It is worth noting that these mid-frequency FOIs, including the FOI from the current study, were higher than the FOIs in the prior studies reviewed above, which involved cooperation or coordination but mostly without verbal communication (Fig. 2), highlighting the role of verbal communication on IBC during cooperative interactions.

The increased IBC in the mid-frequency FOI could also be attributed to motor synchrony, occurring at the pace of 2–4 s, especially for Experiment 2 and Experiment 3, both of which involved explicit dyadic cooperation between participants. Specifically, mother–child dyads participated in a map task in Experiment 2 in two conditions, and dyads of college students performed a dual-task paradigm with the primary task of playing Jenga in Experiment 3. During both Experiments, the synchronized movements of fingers, hands, arms, and bodies as participants take turns performing the tasks (e.g., pointing to the map, holding a block), as well as observing these actions from their partner, may lead to elevated synchronizations between two brains due to the activities of mirror neurons. In line with results in the current study, our previous study^8^, which involved school-aged children playing a Jenga game and employed a bin-by-bin data-driven analysis method, also reported an FOI of [0.29, 0.35] Hz. In contrast, motor synchrony may be less probable between teacher-student triads during math instruction in Experiment 1.

Finally, the increased IBC within this FOI could also be due to Respiratory Sinus Arrhythmia (RSA), occurring around 0.12–0.45 Hz. RSA refers to heart rate variability in synchrony with respiration due to rhythmic changes in cardiac parasympathetic activity^85^. RSA synchrony has been observed during rest and is task dependent, with greater changes in the synchrony associated with tasks that involve challenging behavior and emotion regulation^86^. In the developmental literature, mother–child RSA synchrony is believed to be closely related to children’s brain maturation and ability to form interpersonal attachments, self-regulate, and engage positively with their environment^87^. Likewise, RSA synchrony has been studied in romantic relationships to understand its association with relationship functioning^88^. Interestingly, greater IBC in the regular channels than in the short channels was also observed in this mid-frequency FOI during rest in Experiment 1. Taken together, these results suggest that verbal communications and motor behaviors may foster synchronization between interaction partners through modifying their RSA activities, manifested as synchrony in largely comparable FOIs across the three experiments. We have to acknowledge that this theory cannot be tested with the data available in the current study; future studies may test this hypothesis by monitoring the brain and electrocardiogram signals simultaneously during fNIRS hyperscanning sessions.

The low-frequency FOIs of [0.07, 0.16] Hz across the three studies are below the range of respirational signals but overlap with the Mayer waves, defined as oscillations of arterial pressure occurring spontaneously in conscious subjects^77^. Mayer waves are amplified during sympathetic activation and relatively consistent within the same species. The result that participants showed increased IBC in this range across studies with and without interaction was not surprising; it points to the effect of mere presence on the synchronization between two partners^7^. This FOI ([0.07, 0.16] Hz) also overlaps with the mean frequency across all the FOIs reviewed above (Fig. 2). Excluding the three studies that examined frequencies up to 4 Hz^83,84^ and the study that specifically focused on the IBC between heartbeats^83^, the mean values and standard error of the mean (SEM) frequencies across the identified FOIs across the 93 studies were 0.14 Hz and 0.01 Hz, respectively, with the mean range of the FOIs being 0.16 Hz. We suspect that the low-frequency FOIs were driven by the slow concentration changes in hemoglobin from the cerebral tissues associated with neuronal activity through neurovascular coupling. Finding analogous activities across three experiments in the current study, each involving different social interactions among diverse groups, suggests the presence of shared mechanisms that align multiple brains and support general social interactions. This hypothesis should be explored in future research.

The very-low frequency FOI of [0.02, 0.04] Hz suggests similarities in the slow changes in fNIRS signals. This frequency range has been reported in multiple studies that used data-driven analyses and the reverse of task durations, i.e., 25–50 s (Fig. 2). Increased IBC in this frequency band has been proposed to be associated with prolonged eye contact during social interaction^89^, which was often not observable when participants had their eyes closed. Because this FOI captures very slow neural activity, significant IBC implies that participants’ brains were synchronized over a sustained duration, such as 25–50 s. Possibly due to this reason, the IBC results in this IBC varied a lot across sample sizes (Fig. 6) and populations (Table 1).

Findings from the current study shed light on our earlier question of how social interactions may influence the synchronization of systemic signals, including heart rates, parasympathetic nervous signals, and Mayer waves between different brains. We would like to highlight that the FOIs may be different in the few prior studies that used tasks that were comparable with those in the current studies, due to the different methods used to determine FOIs. For instance, in two studies involving dyadic cooperation, different FOIs were mentioned based on data-driven analyses^83,84^. Between the two studies, this variation could be attributed to the differing experimental designs: one study employed a mixed design^83^, while the other used a within-subject design^84^, thus complicating the comparisons of IBC results. This example exemplifies how different methodological choices for determining FOIs can lead to varying outcomes.

### Effect of channel exclusions and sample sizes on the calculation of IBC

Given the poor signal-to-noise ratios of fNIRS data, which can be further compromised by hair artifacts or inadequate contact between the fNIRS optodes and the skin, we investigated how fNIRS signal quality and channel exclusion influence the calculation of IBC. We calculated the SCI—the correlation between heartbeat signals recorded in the two wavelengths per channel—to indicate the signal quality^90^. However, prior studies have used different SCI cutoff thresholds, leading to various portions of data being excluded. Some studies adhered to the initial recommendation by using an SCI cutoff threshold of 0.75^90,91^. Others preferred a more conservative approach, excluding minimal channels and selecting a lower SCI cutoff threshold^92,93^. A few studies chose an intermediate SCI cutoff threshold to ensure a certain number of short channels included for further analysis^7,79,94,95^. Our prior study demonstrated that both lower (SCI = 0.15) and higher (SCI = 0.75) SCI cutoff thresholds yielded comparable statistical results in single-person fNIRS research with well-controlled block-design stimulation. We must highlight that variations in signal quality can cause the same SCI cutoff threshold to result in different proportions of channels being excluded.

To date, no study has investigated the impact of channel exclusion on the calculation of IBC results in fNIRS hyperscanning research, which often occurs in ecologically valid settings. Our results (Figs. 5 and 6) revealed that channel exclusion affected the statistical results more when the sample sizes were relatively small. When the sample sizes are sufficient, such as 32 for studies involving dyadic settings (Experiment 2 and Experiment 3), channel exclusion rates may affect the statistical results for the mid-frequency ([0.22, 0.49] Hz) and low-frequency FOI ([0.073, 0.17] Hz) less. In contrast, the very-low frequency FOI (under 0.04 Hz) results varied a lot across various sample sizes and various exclusion rates. Across three FOIs under 0.5 Hz (Table 1), a sample size of 32 or above, with a channel exclusion rate of 5–7% achieved robust results across data obtained from three independent experiments. Differences in signal quality (hence various channel exclusions) and different sample sizes in prior studies could have contributed to inconsistent results across studies using a similar paradigm and methodology to determine FOI.

### Limitations of the current method

The proposed methodology offers the advantage of addressing the neural origins of inter-brain synchronization during social interactions in ecologically valid settings, while circumventing the circularity issues often observed in the bin-by-bin frequency analyses as discussed above. We must acknowledge at least four limitations of the proposed method. First, we were able to utilize short-channel detectors from NIRx devices, which are among the most popular for fNIRS research. However, not all commercially available fNIRS systems include short-channel detectors, limiting the generalization of this methodology. Second, to select the FOI, we focused on the WTC method, which is the most popularly used method in the literature, when computing the IBC between two or more individuals. Future studies that are interested in other metrics such as Pearson correlation may similarly select FOI by comparing the correlations between the regular and short channels across frequency bins to identify the frequency bins that show more significance in the regular channels. In addition, as the three datasets involved in the current study were acquired using the same type of devices with the same sampling frequency, we did not examine whether varying sampling frequencies (varying frequency resolution) affect the FOI results based on the WTC method. Future studies can thoroughly test our method with data collected using different devices (with poorer or better frequency resolutions) and various task durations. Third, while the method can reduce arbitrariness in the choice of FOI to improve reproducibility of future research findings in the fNIRS literature, variations in the FOIs between three experiments involving different paradigms and populations still exist. Fourth, the participant pool for all three studies was exclusively Asian. Since darker, denser hair attenuates light more effectively, this can introduce greater artefacts (and relatively higher ratios of channel exclusions) in fNIRS data compared to data acquired from individuals with light-colored hair. Further research is needed to confirm these findings in individuals with light-colored hair.

## Conclusion

Previous fNIRS hyperscanning studies have reported varied selections of frequency bands of interest (FOIs), potentially contributing to inconsistent findings of inter-brain coherence (IBC). In the current study, we briefly reviewed the different methods previously used to determine FOIs in studies focused on cooperation and coordination and discussed the associated challenges. We then proposed a method including short channels to identify FOIs and to address the neural origins of inter-personal brain synchronization. Testing this method on three independent datasets, we found that three datasets identified comparable yet slightly different FOIs that showed greater IBC between different populations and across various social interaction contexts. The shared and unique FOIs of signals driving the IBC between two or more brains could provide a common ground for the comparisons of IBC results in future hyperscanning studies. Our findings further revealed the effect of fNIRS signal quality (hence channel exclusion) and sample size on detecting FOIs and calculating IBC. Future studies may consider reporting data quality and detailing the number of excluded channels to enhance the transparency and reproducibility of the research results.

## Supplementary Information

Below is the link to the electronic supplementary material.

Supplementary Material 1

## Data Availability

MATLAB code used for the data analyses is stored on Open Science Framework and available for peer review through the following link https://osf.io/r4s73/?view_only=6c14eb3ca218417489e939bbd45f7197. The summary (de-identified) data supporting the conclusions of this article will be made available by the authors, without undue reservation.
