# Supplementary material for: Addressing arbitrary choices of frequency band of interest in fNIRS hyperscanning
Source: Sci Rep. 2026 Apr 27;16:19400. doi: 10.1038/s41598-026-50540-z (PMC13287691; doi:10.1038/s41598-026-50540-z)
Supplement: Supplementary file 1 — Supplementary Material 1 [file 41598_2026_50540_MOESM1_ESM.docx]

**Supplementary materials**

# Three sets of experiments

Datasets from three experiments involving different populations and various social interactions were presented in this study. **In Experiment 1**, triads of an instructor and two third-graders were involved in two math instructional sessions (Fig. S1a). Each experimental session followed a fixed sequence across two mathematical topics (counterbalanced across triads): resting baseline, pre-test, instruction, and post-test. During baseline, participants closed their eyes and minimized movement to reduce fNIRS motion artefacts. Pre- and post-tests were completed individually without peer interaction. In **Experiment 2**, mother-child dyads played a map task under two conditions (Fig. S1b). In the first condition, children played a map task independently; in the second condition, mothers were invited to join their children and interact with them as they would at home. In **Experiment 3**, dyads of two college students cooperatively played Jenga games—primary task, while listening to Cantonese stories—secondary task (Fig. S1c).. We manipulated the secondary task by instructing participants to either attend to the stories and be expected to answer story-related questions at the end (i.e., active listening) or ignore the stories and focus on the Jenga game (i.e., passive listening). The stories were presented with background noises at a relatively Easy or Hard levels. In total four conditions were presented, i.e., active vs. passive listening with Easy vs. Hard noise level, with the order of conditions counterbalanced across dyads. In addition, after completing each session, participants also completed a National Aeronautics and Space Administration-Task Load Index (NASA-TLX) survey.


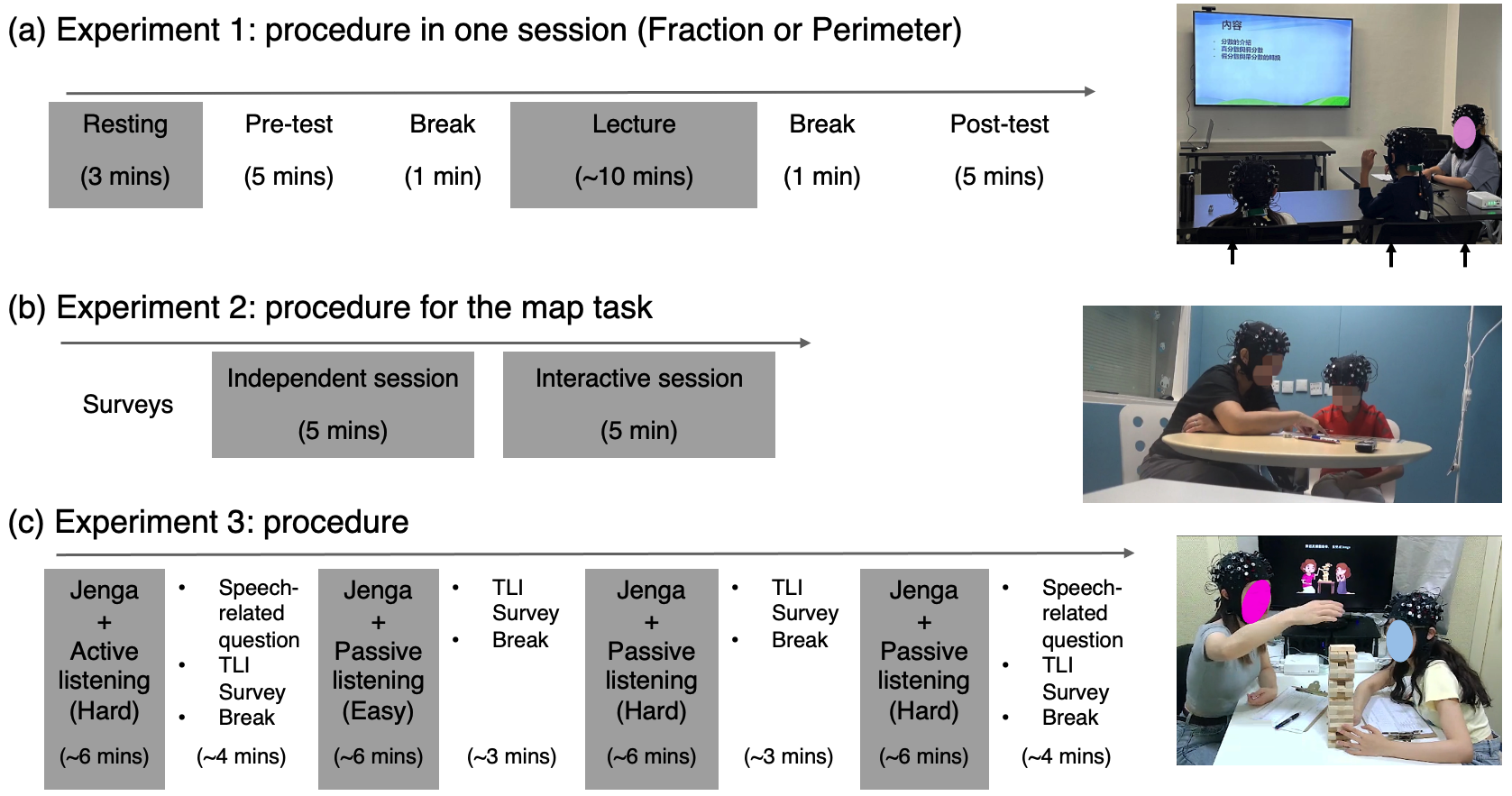


**Fig. S1** Study paradigms for the three experiments.

# Channel exclusion and sample size on the selection of FOI in Experiment 1

## We investigated the effect of channel exclusion and sample size on the selection of frequency bands of interest (FOIs) in section 3.2 in the main manuscript. Fig.S2 and Fig. S3 plot the results for data in Experiment 1 (mathematics learning).


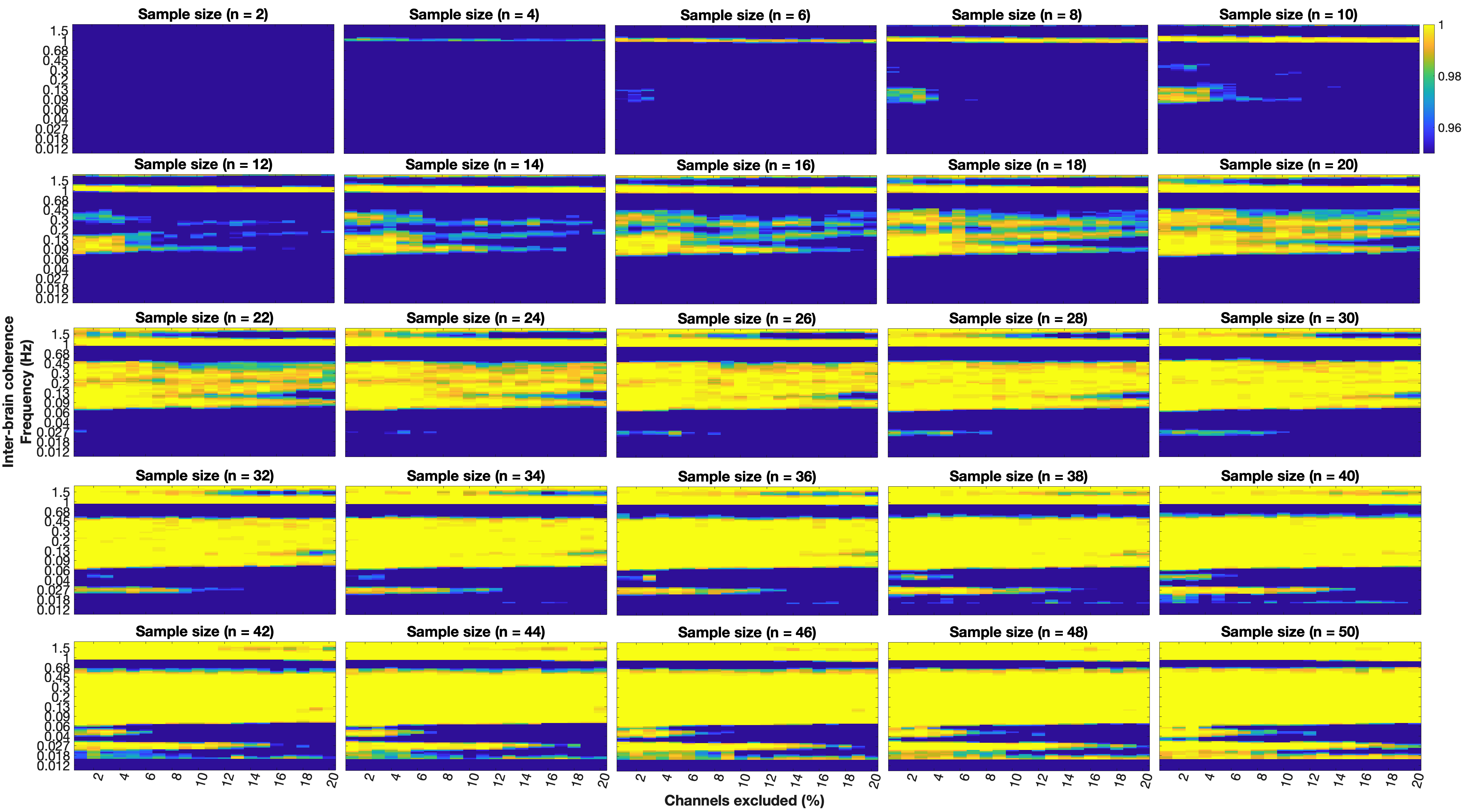


**Fig. S2** Significance of frequency band of interest (FOI) across various channel exclusion rates and sample sizes. This plot was based on data from Experiment 1. Yellow colors indicate robustness above 95% among 500 repetitions.


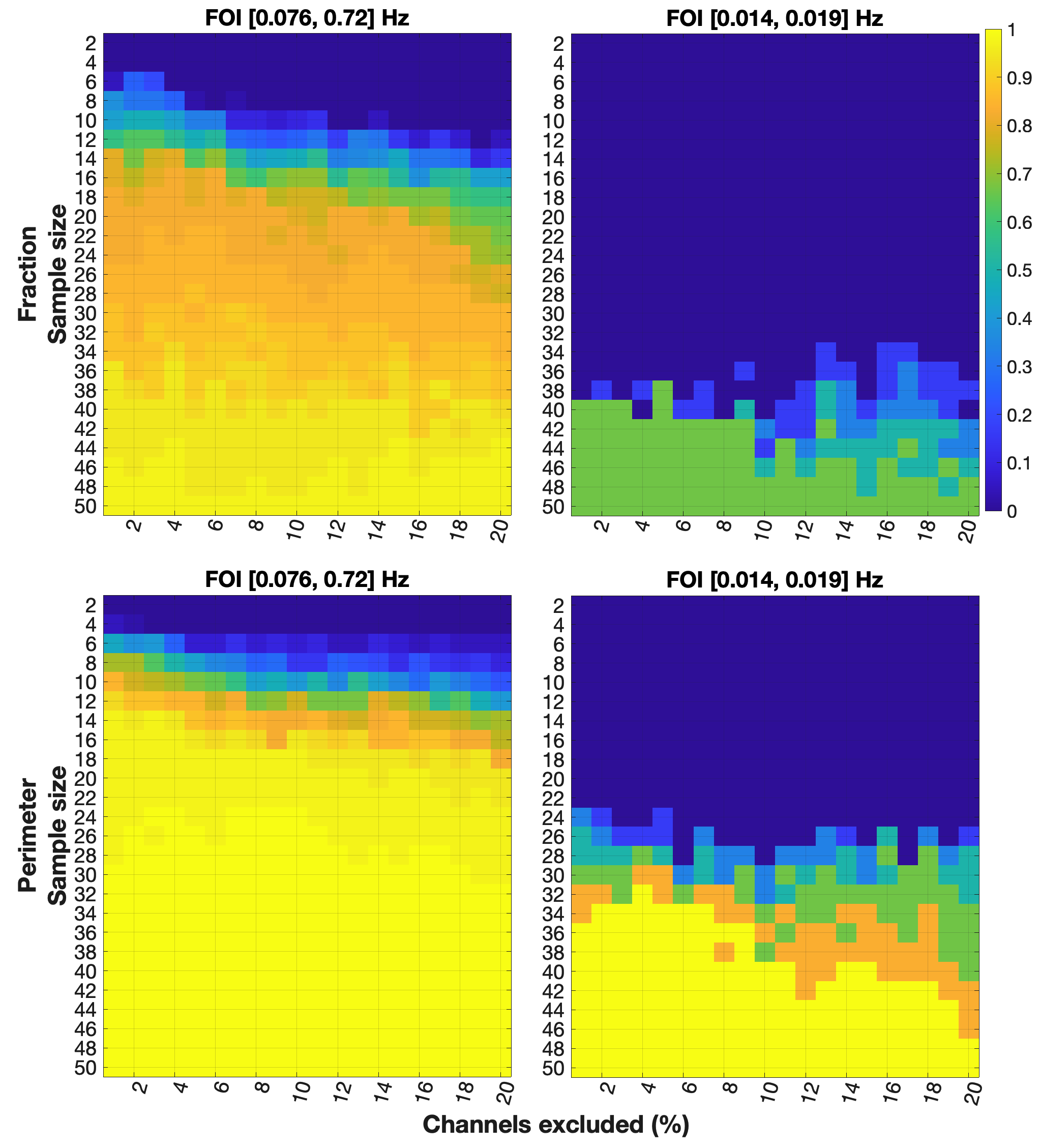


**Fig. S3** Significance at the FOI level. Panels (A) and (B) plot the results from the Fraction and Perimeter lecture sessions in Experiment 1, respectively.

# Inter-beat-interval coherence

Heartbeat is the dominant signal in fNIRS data, and exists in both the extracerebral (superficial) and cerebral (deeper) tissues. However, prior studies have often overlooked frequencies above 1 Hz (heartbeat) when examining inter-brain coherence (see our review in the manuscript). Little is known about the cardiac dynamics during social interactions. To fill the gap, we analyzed heart rate (the number of heart beats per minute) data for dyads of college students in **Experiment 3**. We extracted the heartbeat information and calculated inter-beat-interval from fNIRS signals per channel for individuals, following the procedure described in a prior study^1^, with MATLAB code provided by the authors. We then computed the inter-beat-interval coherence between dyads of participants using the wavelet transform coherence (WTC) method across 92 frequency bins. We compared the inter-beat-interval coherence between the regular and short fNIRS channels, following the same philosophy as the analysis of inter-brain coherence (section 2.3). Finally, we examined the effect of channel exclusion and sample size on the frequency bins for showing significant inter-beat-interval coherence. Our results found that in three frequency clusters, i.e., [1.71 1.92] Hz, [0.090 0.101] Hz, and [0.045 0.050] Hz, the regular channels showed greater inter-beat-interval coherence than the regular channels. Two frequency clusters (i.e., [1.71 1.92] Hz and [0.090 0.101] Hz) overlap a bit with two FOIs (i.e., [1.03 2] Hz and [0.073 0.163] Hz) in Experiment 3 that showed significant IBC in the regular versus short channels. As the two FOIs correspond to the heartbeat and Mayer waves, respectively, these results suggest an increase in synchrony in both neuronal responses and associated cardiac activity within the cerebral issue between two interactive participants during cooperative hyperscanning.


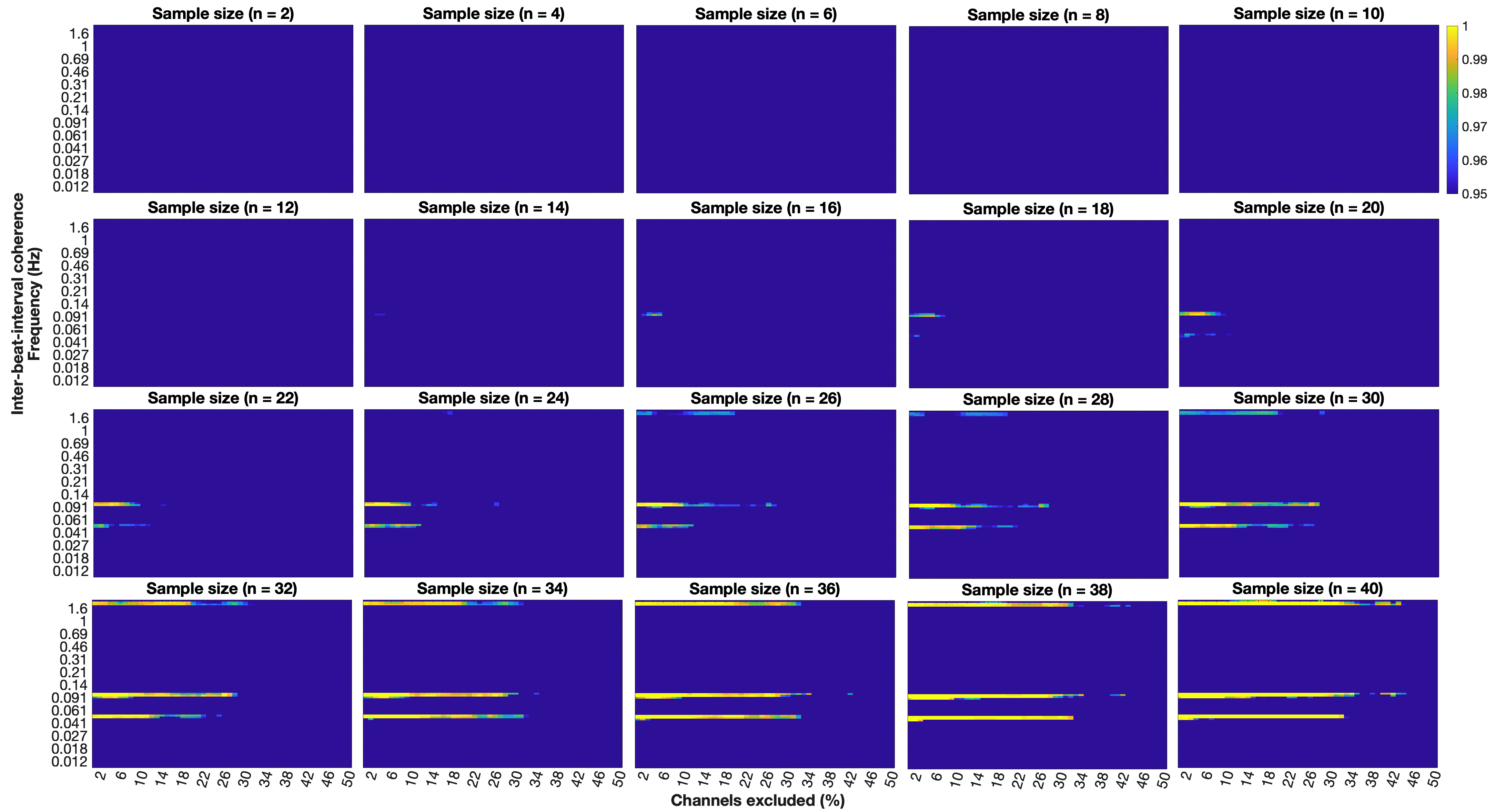


**Fig. S4** Significance of inter-beat-interval coherence across various channel exclusion rates and sample sizes. This plot was based on data from Experiment 2. Yellow colors indicate robustness above 95% among 500 repetitions.

1 Shoushtarian, M., Weder, S., Innes-Brown, H. & McKay, C. M. Assessing hearing by measuring heartbeat: The effect of sound level. *PloS one* **14**, doi:10.1371/journal.pone.0212940 (2019).
